# Quinolizidine Alkaloid Composition of White Lupin Landraces and Breeding Lines, and Near-Infrared Spectroscopy-Based Discrimination of Low-Alkaloid Material

**DOI:** 10.3390/plants14213327

**Published:** 2025-10-30

**Authors:** Stefania Barzaghi, Barbara Ferrari, Elisa Biazzi, Aldo Tava, Paolo Annicchiarico

**Affiliations:** Council for Agricultural Research and Economics, Research Centre for Animal Production and Aquaculture, 26900 Lodi, Italy; stefania.barzaghi@crea.gov.it (S.B.); barbara.ferrari@crea.gov.it (B.F.); elisa.biazzi@crea.gov.it (E.B.); aldo.tava@crea.gov.it (A.T.)

**Keywords:** discriminant analysis, genetic resources, landraces, *Lupinus albus*, NIRS, non-destructive sample, seed quality

## Abstract

White lupin improvement is challenged by the need to select for low seed content of total quinolizidine alkaloids (QAs) when crossing low-alkaloid (sweet-seed) with bitter-seed (landrace) material. This study, which focused on 45 international landraces and 142 broadly sweet-seed breeding lines, aimed at (a) assessing the ability of Near-Infrared Spectroscopy (NIRS) to distinguish broadly sweet-seed from bitter-seed material and, possibly, lines with particularly low QA content within broadly sweet-seed material; and (b) comparing landrace and breeding material in terms of the composition and amount of QA compounds. QA content was analyzed using a gas chromatography–mass spectrometry method. NIRS analyses were performed either on whole-seed samples or ground samples. The range of variation for total QA was 95–990 mg/kg among breeding lines and 14,041–37,321 among landraces. NIRS was able to discriminate broadly sweet-seed from bitter-seed material when using flour samples, non-destructive 10-seed samples, and even individual whole seeds (with <1% misclassification). It was unable to identify material with particularly low QA content. Landrace and breeding line germplasm differed in the proportions of individual QAs. Patterns of geographical variation for total QA content of landraces were identified. Our results can contribute to define an efficient NIRS-based pipeline to select for low total QA content.

## 1. Introduction

White lupin (*Lupinus albus* L.) is a grain legume with a long history of cultivation in the Mediterranean region [1]. This crop is receiving renewed attention as a component of functional, nutraceutical, healthy, or vegan food. This is due to (a) its high seed protein content (34–45%) with a good content of essential amino acids and several useful techno-functional properties [2,3]; (b) the positive effects on human health that it can exert with respect to diabetes, hypertension, cardiovascular diseases, and obesity [4,5]; and (c) its seed content of oil (in the range of 8–12%) with excellent nutritional characteristics [6]. In addition, white lupin has high potential interest as a high-protein feed crop for rain-fed environments of Southern Europe [7]. In this region, it exhibited higher protein yield per unit area than other cool-season grain legumes such as pea, faba bean, and narrow-leafed lupin [8]. However, selecting varieties with greater yielding ability is considered indispensable to boost the cultivation of this crop [9].

White lupin genetic resources are limited to the primary gene pool [1]. Landraces are extremely important, as they may display (a) excellent yielding ability in specific regions [10,11]; (b) tolerance to key stresses such as drought [12], low temperatures [13,14], and anthracnose [15,16]; and (c) adaptation to calcareous soils [17,18]. However, their exploitation is severely hindered by their high seed content of quinolizidine alkaloids (QAs). These compounds, which may exceed 30,000 mg/kg in the seed [19], are secondary metabolites synthesized by lupin species and other plants as a defense mechanism against pathogens and herbivores [20,21]. In addition, seed QAs may act as a storage means for nitrogen used by seedlings after germination [22]. QAs confer a bitter taste to lupin seeds and can be toxic for humans and animals [23]. The total QA seed content of modern sweet-seed varieties should ideally be below the threshold of 200 mg/kg that is recommended for lupin-based food according to the Health Authorities of various countries [24,25] and, anyway, below the threshold of 500 mg/kg that is set for use as animal feed [26]. However, the latter threshold may be exceeded frequently by elite breeding lines [27] and occasionally by commercial varieties [28,29]. The breeding of sweet-seed varieties is based on the exploitation of the *pauper* locus [30,31], a recessive gene encoding for an acyltransferase involved in the early QA pathway that has a strong depressive effect on QA biosynthesis [23,32]. This locus, however, does not ensure the achievement of sufficiently low total QA content, owing to (a) the effect of different allelic forms [30]; (b) possible non-allelic gene interactions [33]; and (c) minor genes that generate a complex trait inheritance pattern mirroring the complexity of the QA biosynthetic pathway [23]. To ease the challenge of achieving sufficiently low QA content, white lupin breeders tend to avoid the use of landrace genetic resources in crossing programs. This trend seriously limits crop improvement and restricts the genetic diversity of modern germplasm, causing a domestication bottleneck that was thoroughly investigated in narrow-leafed lupin [34]. Such a trend could be reversed by the availability of quick, low-cost, reliable methods that are able to discard progeny lines with high QA content in early generations.

The selection of sweet-seed inbred lines from crosses between bitter-seed and sweet-seed parent material can currently be performed by methods that are either lengthy from a breeding viewpoint or not sufficiently reliable. The Dragendorff paper test [30], a colorimetric method exploiting a reaction between QAs and the potassium bismuth iodide reactive, needs be performed on adult plants. Molecular markers that tag the pauper locus have been identified [35,36,37], but their use for selection on seedlings requires a high cost per plant. The fluorescence exhibited by whole seeds (i.e., unbroken seeds) with high QA content when evaluated under an ultra-violet lamp [38] provides a quick test that lacks high discrimination power (probably due to interfering substances such as flavonoids). For the sake of completeness, there is also a spectrophotometer-based method [20,39] that lacks any published adaptation to single seeds. Near-Infrared Spectroscopy (NIRS) could represent a valuable alternative tool to select seeds for low QA content, if it was able to distinguish low-QA from high-QA material in a reliable manner. This rapid and low-cost technique is largely adopted for the seed quality improvement of primary metabolites such as protein and oil content [40,41]. Its adoption for the quantification of secondary metabolites is more challenging because of their low concentration. However, satisfactory results were reported for polyphenol content in faba bean [42], isoflavone and phenolic acid contents in red and white clover [43], phenols and phytic acid in cowpea [44], tannins and phytic acid in common bean [45], and other compounds [46]. Importantly, successful applications of NIRS for QA determination were reported for different plant tissues of velvet lupine (*Lupinus leucophyllus* Dougl.) and larkspur (*Delphinium occidentale* Muth.) [47] and for the rhizome of *Coptis* spp. [48] using ground samples. A recent study used NIRS-based estimations of white lupin total QA content, but provided no published reference or other information on the adopted calibration [49] (which was confirmed as unpublished by the article’s corresponding author). Even when NIRS-based quantification was not sufficiently accurate, NIRS spectra may prove useful for the classification of plant material into a high-value and a low-value class based on content of a target metabolite using discriminant analysis [46,50].

NIRS-based quantification or discrimination for total QA amount in white lupin seed could be explored using ground samples or whole seeds. Whole seed-based predictions are non-destructive and have special interest for plant breeding because, while being more challenging, they offer the advantages of allowing the planting of the evaluated material and saving the seed grinding cost [51]. NIRS equipment that allows for the analysis of individual seeds offers the additional opportunity to select on a single seed basis, as reported for primary metabolites of pea [52]. The NIRS-based distinction of broadly sweet-seed material (usually carrying the *pauper* locus) from bitter-seed material could prove highly beneficial for white lupin breeding. The NIRS-based prediction of total QA content within broadly sweet-seed material (aimed to assess the variation in a range of 50 to 1000 mg/kg) would be extremely useful, although probably too challenging for this technique.

This study focused on a set of white lupin landraces sorted out of a described world germplasm collection [11], and a set of broadly sweet-seed breeding lines that originated from crosses of elite landraces with sweet-seed germplasm and underwent preliminary selection for low alkaloid content [53]. This material was analyzed for QA content using a gas chromatography–mass spectrometry (GC/MS) method. The main objective of our study was to assess the usefulness of NIRS-based prediction models for total QA content in two contexts, namely (a) the discrimination of broadly sweet-seed material from bitter-seed material; and (b) the quantification of total QA content within broadly sweet-seed breeding lines on the one hand and bitter-seed landrace germplasm on the other. NIRS analyses were envisaged either on whole-seed samples or ground samples. A second objective of our study was to compare landrace and breeding material in terms of the composition and amount of QA compounds.

## 2. Results

### 2.1. Variation for Quinolizidine Alkaloid Content

Both germplasm types exhibited large variation for total seed QA content (*p* < 0.001). The variation in breeding lines ranged from 95 to 990 mg/kg (Table 1), with only 24% of the lines showing a value below the optimal threshold of 200 mg/kg. The total QA content of the individual landrace accessions ranged from 14,041 mg/kg in one landrace from Italy to 37,321 mg/kg in one landrace from Greece (Supplementary Table S1). On average, landrace germplasm featured about 75-fold greater total QA content than broadly sweet-seed breeding line germplasm (25,613 vs. 338 mg/kg; Table 1). The sharp difference between these germplasm types was confirmed by the over 14-fold greater QA amount of the least bitter landrace relative to the line with the highest QA content (14,041 vs. 990 mg/kg; Table 1).

The analysis of variance for total QA content of landrace accessions revealed significant variation (*p* < 0.001) both among regional germplasm pools and among landraces within germplasm pools. The comparison among germplasm pools revealed a trend towards higher values for landraces from Greece and western regions (Atlantic islands and Iberian countries) and a trend towards lower values for landraces from Italy and south-eastern regions (Near East, Turkey, Egypt, East Africa) (Table 2). Large variation within the germplasm pool was displayed by landraces from Italy, Greece and, to a lesser extent, Turkey and Portugal (Table 2).

Variation (*p* < 0.01) emerged for all of the 13 QAs both among breeding lines and among landrace accessions. Lupanine, which was the main QA, displayed wide entry variation in both germplasm types (Table 1). The mean proportion of this QA was lower in breeding lines than in landraces (61.6% vs. 81.4%: Table 1). The two germplasm types differed in terms of the relative proportions of other QAs. In particular, 13α-hydroxylupanine and 13α-angeloyloxylupanine were moderately represented in breeding lines (averaging 9.6% and 8.4%, respectively), while being minor QAs in landraces (<2%). Conversely, multiflorine was moderately present in landraces (averaging 9.3%), while being a minor QA in breeding lines (1.5%) (Table 1). Also, albine was the third most represented QA in landraces, while being very low and occasionally absent in breeding lines (Table 1).

### 2.2. NIRS-Based Predictions

**Figure 1:**
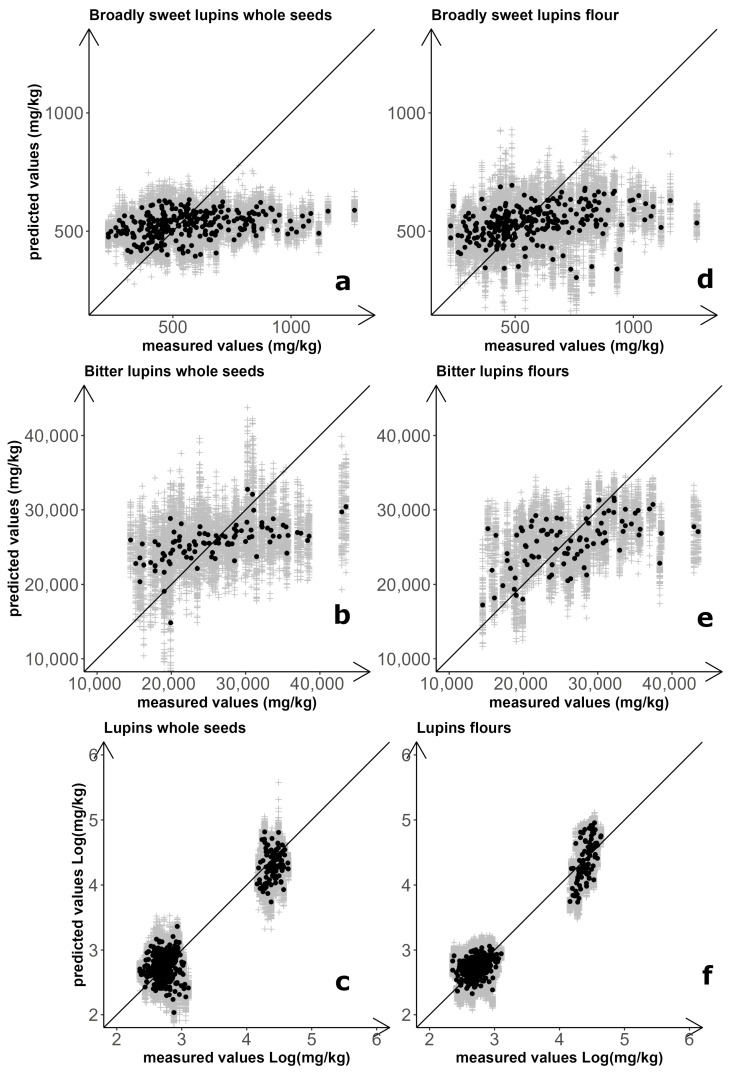
NIRS-based prediction of white lupin total quinolizidine alkaloid content. Results for regression models calculated on both whole-seed and flour spectra of broadly sweet-seed breeding lines (**a**,**d**), landrace accessions (**b**,**e**), and the combination of breeding line and landrace material (**c**,**f**). Grey symbols: results of individual repetitions; black symbols: average of 100 repetitions.

Separately for whole-seed and flour samples, three prediction models for NIRS-based quantification of total QA content were set up and assessed: one for broadly sweet-seed breeding lines, one for landrace accessions, and one for the combination of these materials. Results for the six combinations of sample type and prediction model are summarized in Figure 1 and Table 3. The optimal number of latent variables (LVs) for models focusing on breeding line material were LV = 1 for seeds and LV = 2 for flours. For both sample types, however, the coefficients of determination (*R^2^*) close to zero indicated no prediction ability (Table 3). Improved but still unsatisfactory results were obtained for predictions of whole seeds (*R*^2^ = 0.293) and flour (*R*^2^ = 0.467) of landrace material, using models with LV = 6 for seeds and LV = 2 for flours (Table 3).

Prediction models for QA quantification of the entire set of materials (landrace and breeding lines) were based on logarithmic values of total QA content, to compensate for the huge differences in entry mean and entry variation that occurred between sweet-seed and bitter-seed material. Predictions were satisfactory in this case for both whole-seed samples (*R*^2^ = 0.889) and flour samples (*R*^2^ = 0.932), using LV = 6 and LV = 4, respectively (Table 3). These predictions were satisfactory also according to the Ratio of standard error of Prediction to standard Deviation (RPD), of which an observed value ≥ 3 is considered acceptable for use in quality control applications [54]. However, the improvement in the performance of the calibration models was essentially due to the clear difference in alkaloid content between the two germplasm sets, with inaccurate quantification within germplasm sets that agreed with earlier results (Figure 1). The separation between broadly sweet-seed and bitter-seed material was particularly sharp when based on flour samples, with no overlap of material according to predicted values obtained from 100 repetitions of double cross-validation (Figure 1).

**Figure 2:**
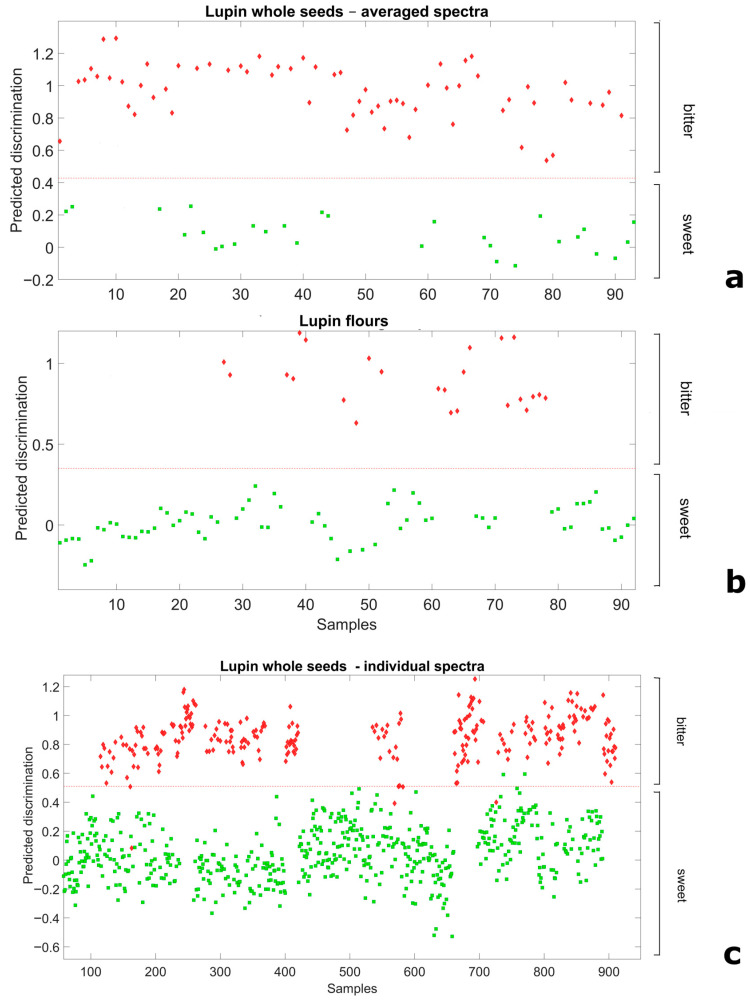
Ability of Partial Least Squares Discriminant Analysis models to discriminate broadly sweet-seed and bitter-seed white lupin material in validation data sets, for (**a**) whole seeds, averaged spectra of 10 seeds; (**b**) flour spectra; and (**c**) whole-seeds spectra of individual seeds (red diamond: actual bitter-seed material; green square: actual sweet-seed material; red broken line: separation based on predicted discrimination).

We explored the scope for NIRS-based distinction of broadly sweet and bitter germplasm using a Partial Least Squares Discriminant Analysis applied on spectral data of whole-seed or flour samples. In the analysis, the material (seed or flour) from breeding lines and landrace accessions was considered a priori as representative of the sweet-seed and bitter-seed classes, respectively, according to results from chemical analyses. For whole seeds, we envisaged not only results based on data averaged across the spectra of the 10-seed sample but also results based on spectra of the individual seeds (also attributed a priori to their relevant class). For both flour and 10-seed average spectra, discriminant models employing only two LVs allowed for a perfect separation of the two classes (Figure 2a,b), as indicated by no misclassification and the unity values of Sensitivity and Specificity in the validation data set (Supplementary Table S2). Remarkably, even the discriminant model relative to 3740 individual whole seeds, which adopted three LVs, achieved an almost perfect separation of sweet-seed and bitter-seed material (Figure 2c). In this case the proportion of misclassified seeds was just 0.88%, whereas the Sensitivity and Specificity values were 0.978 and 0.997, respectively, which yielded a classification error of 0.013 (Supplementary Table S2).

**Figure 3:**
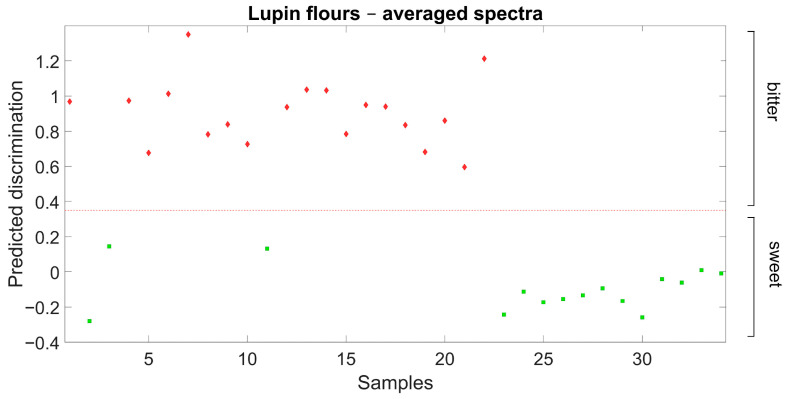
Ability of Partial Least Squares Discriminant Analysis model based on flour spectra to discriminate broadly sweet-seed and bitter-seed white lupin material in an independent germplasm set grown in a different environment (red diamond: actual bitter-seed material; green square: actual sweet-seed material; red broken line: separation based on predicted discrimination).

Further validation results for the discrimination of sweet-seed and bitter-seed flour samples are reported in Figure 3. In this case, the discrimination model previously defined from field-grown material was used for the discrimination of independent sweet-seed and bitter-seed genotypes grown under greenhouse conditions in a different year. The discrimination was also clear-cut in this case, with no misclassification of any observation.

The NIRS spectra obtained from whole seeds exhibited greater dispersion than those obtained from flour (Supplementary Figure S1) due to light scattering effects caused by surface roughness and variability in seed size. This finding could account for two reported results, namely: (a) the greater accuracy of NIRS-based regressions for flour samples relative to whole seeds; and (b) the greater classification accuracy achieved for averaged whole-seed spectra relative to spectra of individual seeds in the discriminant analysis.

## 3. Discussion

The overall range of variation for total QA content in this study (from 95 to 37,321 mg/kg) is close to that reported in the recent evaluation of a large white lupin germplasm collection in Germany (from about 50 to 36,000 mg/kg) [49]. This result confirmed the ability of our data set to adequately represent the variation for this trait that occurs in breeding lines and landrace collections. The current region of evaluation may have produced higher total QA content values than Italian regions featuring a truly Mediterranean climate, based on earlier evaluation studies [29,55]. However, the maximum value of 990 mg/kg of total QA content observed in our breeding lines, although quite high, was lower than that reported for some elite lines from another breeding program [27]. Our study confirmed the difficulty for white lupin breeders to select breeding lines whose total QA content lies below the threshold of 200 mg/kg that is required for direct food use of the grain. Only about 24% of our breeding lines exhibited this characteristic, despite their preliminary selection for low QA content performed on F_3_ and F_4_ individual seeds by two methods, namely, fluorescence and spectrophotometer-based evaluation. The combination of these methods was able to discard bitter-seed material, considering the sharp difference in total QA content between the breeding line with highest QA content and the landrace with the lowest content of QAs. It should be noted that the spectrophotometer-based evaluation, which is expected to be more sensitive than fluorescence but whose reliability is pending verification, is much more time-consuming than a NIRS-based assessment.

**Figure 4:**
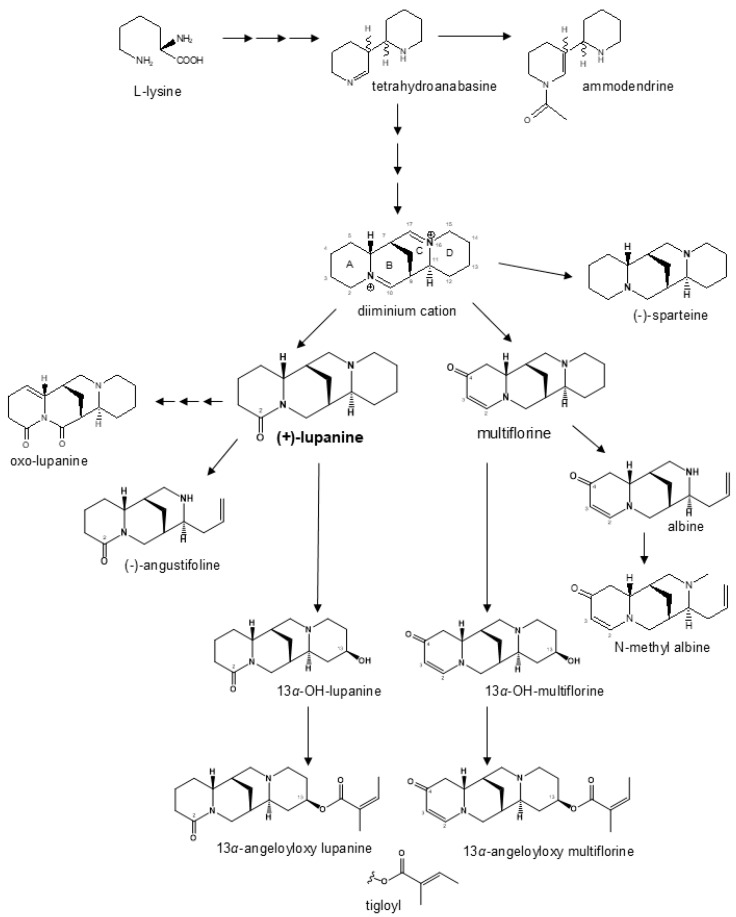
Modified biosynthesis pathway of quinolizidine alkaloids in white lupin based on previously published work [19,21,32].

Breeding line and landrace germplasm groups differed not only for average total QA content (as expected because of the *pauper* locus in the former material) but also for the relative proportion of individual QAs. Lupanine was the main alkaloid in both germplasm groups, but its proportion was higher in landraces than in breeding lines (81% vs. 62%). Multiflorine was the second most represented QA in landrace material while being minor in breeding lines (9.3% vs. 1.5%), in which the other most represented QAs were 13α-hydroxylupanine (9.6%) and 13α-angeloyloxylupanine (8.4%). The biosynthetic pathway of white lupin QAs in Figure 4 shows two main branches, one originating from lupanine and the other from multiflorine. The lupanine branch, including also its derivatives 13α-hydroxylupanine, 13α-angeloyloxylupanine, and 13α-tigloyloxylupanine, appeared to be highly conserved across bitter-seed and sweet-seed materials in terms of average proportion of its QAs, which amounted to nearly 84% in bitter-seed germplasm and about 82% in sweet-seed material. In contrast, the multiflorine branch (including also albine and N-methylalbine) showed a marked reduction in the proportion of its QA compounds in sweet-seed lines, which amounted to 5.3% compared with 12.4% in bitter-seed germplasm. Notably, early intermediates such as multiflorine and albine were reduced, on average, by 466- and 565-fold, respectively, in sweet-seed material relative to bitter-seed material; while the final product, N-methylalbine, had just a 28-fold decrease. While the lupanine pathway is conserved across the *Lupinus* genus, the multiflorine branch appears to be specific to white lupin [56]. Our results suggest that the *pauper* locus has a particularly strong effect on the multiflorine pathway, probably affecting the regulation of an initial step responsible for the production of this alkaloid. On the whole, our findings on QA proportions agree with those for sweet-seed material in [29]. In two earlier studies of white lupin collections largely including bitter-seed germplasm, lupanine was the main QA compound (70–76% averaged across entries), followed by 13α-hydroxylupanine (around 8%), albine (4–15%), and multiflorine (3–5%) (while 13α-angeloyloxylupanine was not assessed) [56,57]. Albine, currently present in a minimal amount or absent in sweet-seed material, was absent in various Australian sweet-seed cultivars [58]. The relatively high average content of 13α-hydroxylupanine in our breeding lines is positive, considering that this compound is less dangerous than other QAs for human and animal feeding [59]. Anyway, QA safety thresholds refer to the total content of QAs.

The total QA content of landrace accessions observed in this study suggested a pattern of geographic variation leading to higher values in material from Greece and western regions (Atlantic islands and Iberian countries), and lower values in material from Italy and south-eastern regions (Near East, Turkey, Egypt, East Africa). Concurrently, the two countries in the central Mediterranean region, Italy and Greece, exhibited particularly large variation for this trait. Earlier evaluations of regional landrace pools (which included a lower number of evaluated QAs) anticipated some of the current findings, such as the trend towards high QA content of landraces from the Azores [28,29], the trend towards low values of landraces from Egypt [28] and the Near East [29], and the large variation for total QA content of landraces from Greece [28] and Italy [29]. The current information on the total QA content of regional landrace pools can be useful for breeders to identify useful genetic resources in breeding programs. Using landrace genetic resources with lower QA content produced breeding lines selected for the *pauper* locus that showed lower QA content than lines whose landrace parent germplasm featured high QA content [60].

The total QA content of white lupin is influenced by several environmental factors [23,59] but is not affected largely by genotype × environment interaction, which was non-significant for a large white lupin collection evaluated across German environments [49] and distinctly smaller than genotype main effects for breeding lines in Italy [55]. The modest extent of genotype × environment interaction reinforces the reliability of the reported results for landraces, and facilitates the selection for low total QA content.

NIRS-based discrimination has mostly been proposed for the classification of species or geographic origins of plants and for plant quality assessment [46]. The results from this study encourage the adoption of NIRS as a quick and reliable method for the discrimination of broadly sweet-seed material from bitter-seed material within segregating progeny lines derived from crosses between sweet-seed and bitter-seed (landrace) parent plants. This proved possible not only according to NIRS predictions based on a flour sample, which is a destructive sample of pooled progeny seeds from a test plant, but also according to predictions based on a non-destructive, whole-seed sample. Using a whole-seed sample eliminates seed grinding costs and allows breeders to sow the seed evaluated by NIRS (rather than other stored seed). The latter advantage can be important when evaluating individual plants whose seed production is limited by unfavorable growing conditions. Our results indicated an additional opportunity for NIRS-based discrimination that has extreme practical interest for breeders, i.e., that relative to individual whole seeds. This opportunity, which is supported by the results of the discriminant analysis in Figure 2c, allows breeders to select individual plants prior to their sowing in early generations (e.g., F_3_ or F_4_), when the progeny seed of one plant is a mixture of broadly sweet-seed and bitter-seed genotypes and lacks sufficient genetic homogeneity for a meaningful selection based on a pooled seed sample (such as the current 10-seed one). Sufficient genetic homogeneity for a convenient NIRS screening based on a pooled seed sample would be reached only in later generations obtained by single-seed descent (e.g., F_5_ or F_6_). The advantage of a timely screening on individual whole seeds in early generations would largely counterbalance the small rate of seed misclassification (<1%) observed for NIRS discrimination in this case. A limitation of our study was the lack of validation of the discriminant analysis model for individual whole seeds, which was technically prevented by insufficient material made available by single seeds for the chemical analysis. In addition, our validation work on independent material grown in other environments was limited to flour samples of one data set, and would require further assessment. The generation of additional data (which must cope with the high cost of the chemical analysis) could also refine and improve our NIRS-based prediction models.

The good ability to discriminate broadly sweet-seed from bitter-seed materials showed by NIRS exploited the sharp difference in total QA content between these materials. Conversely, NIRS regressions aimed at the quantification of total QA content were not sufficiently accurate for landrace material, and were totally inaccurate for the most challenging scenario of predicting material with very low total QA content within broadly sweet-seed material. This last result could be attributed to the very low concentration of QAs in sweet-seed materials. NIRS is best suited to quantifying major, abundant components, and the signal produced by QAs in this material was weak and could easily be masked by noise or overlapping peaks from other components. Furthermore, the functional groups in alkaloids (such as the nitrogen-containing rings) do not contribute significantly to the NIR spectrum, making it difficult to detect them. Therefore, other means are needed by breeders for selecting lines with very low QA content. Besides chemical analysis, which entails high costs, a promising means is represented by genomic selection. A recent study focusing on the current set of breeding lines revealed a predictive ability (as the correlation between genomically modeled and observed data) of 0.66 for total QA content, using molecular data issued from genotyping-by-sequencing [60]. While pending verification from a wider set of breeding lines and/or evaluation environments, genomic selection would imply lower costs than chemical analysis based on the current GC/MS method. In addition, its genotyping data could contribute at virtually no extra cost to genomic selection for other important traits, such as (a) grain yield in specific growing regions [61], under severe drought [53] or in moderately calcareous soils [62]; (b) tolerance to anthracnose [49]; and (c) other seed quality traits [49,60].

In conclusion, this study generated novel scientific information in two respects. On the one hand, it reported on differences in the extent and composition of QA compounds between and within bitter-seed germplasm and sweet-seed germplasm possessing the *pauper* locus. These indications could assist breeders in locating landrace sources with lower QA amounts, and contribute to clarify the effect of the *pauper* locus on QA metabolic pathways. On the other hand, it devised a NIRS-based method that could contribute to define an efficient pipeline for white lupin selection for low total QA content of breeding lines derived from crosses between sweet-seed and landrace germplasm. Upon further verification of its high reliability on whole-seed samples of independent breeding lines grown in other environments, this rapid and low-cost method could ease the utilization of crucial genetic resources and the broadening of the genetic diversity of cultivated material. In particular, very large numbers of individual seeds of early segregating material, such as that of the F_3_ or F_4_ generations, could undergo a two-stage selection of broadly sweet-seed material, first via a very quick—albeit somewhat inaccurate—method such as fluorescence [38], and then via NIRS-based discrimination. Selection for very low total QA content would occur in later generations on promising inbred lines, possibly through genome-enabled predictions for this [60] and other important traits.

## 4. Materials and Methods

### 4.1. Plant Material

Our study focused on two germplasm sets that provided whole-seed and flour samples for NIRS predictions and underwent chemical analyses of total and individual QA contents. The first set was composed of 142 broadly sweet-seed breeding lines originating from crosses between each of four elite sweet-seed cultivars or breeding lines with each of four elite landrace accessions. Details on the development of the breeding lines are provided in [53]. The sweet-seed parent material comprised the varieties Lucky from France and Arsenio from Italy and the breeding lines L27PS3 from Morocco and MB-38 from France (all possessing the *pauper* locus). The landrace parent germplasm included the accessions LAP123 and La246 from Italy, La646 from the Canary Islands, and Gr56 from Greece. The inbred lines were obtained by single-seed descent under insect-proof cages (to prevent cross-pollination), with the aim of generating a similar number of lines from each of the 16 crosses. Within-cross selection for low QA content was performed (a) on F_3_ and F_4_ individual seeds using the fluorescence method [38], and (b) on F_4_ seeds using a non-destructive test whose ability to predict the total QAs content of progeny plants is pending verification. This test, which consisted of a method published in [20,39] adapted to single seeds, involved the following stages: (a) immersion of one weighed seed in 3 mL of deionized water for 24 h; (b) spectrophotometer-based determination of the alkaloid content in the water using Reifer’s reagent (14% KI, 9% I2 in distilled water) at 830 nm, using sparteine for calibration; and (c) the expression of this content in ppm of seed dry weight. The test was used to discard material whose predicted QAs content belonged to the highest 25% quartile. The final population included 960 F_6_ inbred lines, of which 142 lines were chosen for this study.

The second germplasm set included 45 landrace accessions selected from a world collection previously described [11]. These accessions, which are listed in Supplementary Table S1, belonged to 11 germplasm pools representing major historical regions of white lupin cultivation, namely Greece, Italy, Egypt, Turkey, Spain, Portugal, Azores, Canary Islands, Maghreb (including Algeria and Morocco), East Africa (including Ethiopia and Kenya), and West Asia (including Syria, Lebanon, Israel and Jordan).

Seed samples for the two germplasm sets were generated from two nearby field experiments, one for breeding lines and the other for landrace accessions. The experiments were performed in Lodi (northern Italy; 45°19′ N, 9°30′ E) according to a randomized complete block design with two replications. They were sown in mid-October 2018 and harvested in late June 2019. Each plot comprised 30 plants disposed in three rows spaced 0.30 cm. The seed was inoculated with Vitalianz R Lupin inoculant (Cérience, Cissé, France) prior to sowing. The experiments featured 57 frost days and a lowest absolute temperature of −12.0 °C and received 525 mm of rainfall over the crop cycle. Each plot provided a random sample of 10 seeds that was used for NIRS and chemical analyses.

An additional, small set of lupin genotypes that included 19 individual plants sorted out from the set of landrace accessions (considered as bitter-seeded) and 15 plants sorted from commercial varieties (considered as sweet-seeded) provided an independent validation data set of flour samples. These plants were grown in a non-heated greenhouse in Lodi from October 2017 to June 2018, under moisture-favorable, mild-winter conditions with a lowest absolute temperature of −3.0 °C.

### 4.2. NIRS-Based Evaluation of Quinolizidine Alkaloids

A set of 10 whole seeds per plot was analyzed by NIRS using a NIRFlex 500 spectrometer (Büchi Italia, Cornaredo, Italy). The spectra were recorded in reflectance mode across the spectral range of 10,000 to 4000 cm^−1^, recording data for the individual seeds by employing the 10-seat tablet adapter of the measurement cell. The spectra of the 10 individual seeds were averaged for prediction purposes. Subsequently, each 10-seed sample was milled using a MM400 Mixer Mill (Retsch Gmbh & Co., Düsseldorf, Germany) at 30 Hz for about 40 s. The resulting flour samples were also analyzed using NIRS, employing the measurement cell vial adapter with the same spectral range used for seeds. To improve the NIR spectral reproducibility of the flour replicate samples, three scans were performed for each plot sample after re-blending the sample in the vial between each scan, averaging the spectral data of the three replicate scans.

### 4.3. GC/FID and GC/MS Analysis of Quinolizidine Alkaloids

The extraction and quantitation of QAs was performed in triplicate on each plot flour sample according to a modified version of a protocol described in [29,57]. Details of this protocol are provided elsewhere [63]. In brief, 50 mg of defatted flour was suspended in 1.2 mL of 0.1 N HCl, with sparteine (CAS 90-39-1; Extrasynthese, Genay, France) added as internal standard in an appropriate concentration and stirred at room temperature overnight. The mixture was centrifuged at 8000× *g* for 45 min at 4 °C, the supernatant was collected, and the solid was washed twice with 0.8 mL of 0.1 N HCl. The gathered extracts were treated with 5% NH_4_OH to pH 10–11 and then applied onto an Extrelut NT 3 column (Merck, Darmstadt, Germany). The alkaloids were eluted with CH_2_Cl_2_ (4 × 3 mL), and the solvent was evaporated under vacuum. The residue was diluted in CHCl_3_ and analyzed by Gas Chromatography with Flame Ionization Detector (GC/FID) and GC/MS. The identification of the QAs was performed by GC/MS; quantitative evaluation of the QAs was determined by GC/FID using the internal standard methods.

A Perkin-Elmer Clarus 500 GC equipped with a capillary column Elite-5 MS (DB-5, 30 m × 0.32 mm × 0.25 μm; Perkin-Elmer; Milan, Italy) was used for GC/FID analyses, operating at the following conditions: oven temperature 90 °C for 2 min, increased to 300 °C at 7 °C/min, and held at 300 °C for 10 min. Helium was the carrier gas, at a flow rate of 2 mL/min. Injector and detector temperatures were set at 300 °C and 320 °C, respectively. Samples (1 μL) were injected in ‘splitless’ mode. The response factor of GC/FID was calculated using the ratio between the response of the internal standard (sparteine) and the response of the analyte standard lupanine (kindly provided by Prof. M. Wink). The regression coefficient between the analyte concentration and detector response was *R*^2^ = 0.99. A Perkin Elmer Clarus 500 GC equipped with a Clarus 500 mass spectrometer was used for GC/MS analyses, using the same capillary column and chromatographic conditions as for the GC/FID. Mass spectra were acquired over a range of 40–400 atomic mass units (amu) at 1 scan/s, with ionizing electron energy of 70 eV and ion source at 230 °C. The transfer line was set at 300 °C and the carrier gas was helium at 1.0 mL/min. The QAs were identified by determination of their elution times from the published mass spectra as reported in [29,57], as well as by a peak-matching library search [64]. This method allowed us to identify 13 QAs, which are listed in Table 1 along with their quantitative evaluation obtained via GC/FID analyses. The total content of QAs was obtained by summing up the quantitative data of the individual QAs.

### 4.4. Statistical Analyses

The occurrence of variation for total QA content and individual QAs was assessed separately for breeding lines and landrace accessions by analysis of variance performed on plot-derived samples. A second analysis of variance limited to landrace germplasm data, which included the fixed factor germplasm pool and the random factor accessions within the germplasm pool, aimed to compare the 11 regional germplasm pools for total QA content. Differences between germplasm pools (which were based on a variable number of accessions) were assessed using paired comparisons through the option PDIFF of the PROC GLM of the SAS/STAT^®^ software version 9.2 [65].

NIRS predictions for total QA content of whole-seed or flour samples were developed by Partial Least Square Regression (PLSR) models calculated on the average spectra using the R package Chemometrics version 1.4.4. The analysis adopted the repeated double cross-validation method [66], which is recommended to identify the optimal number of latent variables and obtain a more reliable estimation of the prediction performance. The double cross-validation was repeated 100 times with different random splits into test and calibration sets. The prediction ability was assessed using *R*^2^ and RPD statistics [54].

Calibration models for the classification of whole-seed or flour samples into broadly sweet seed or bitter-seed classes were developed using Partial Least Squares Discriminant Analysis. These models were calculated using the PLS Toolbox software (ver. 9.3, Eigenvector Research, Inc., Manson, WA, USA). The analysis was performed for three sets of spectral data relative to (a) whole seeds with averaged spectra of the 10-seed sample; (b) flour spectra; and (c) whole seeds spectra of 3740 individual seeds (relative to two replicates of 10 seeds each for 142 breeding lines and 45 landrace accessions). The analyses assumed the actual classification classes of broadly sweet-seed for the breeding line material and bitter-seed for the landrace material, as supported by results of the chemical analysis. They held 75% of the samples for calibration and 25% for validation, using the Duplex algorithm. The spectra were pre-processed using the Savitzky–Golay first derivative. In addition, the discrimination model defined from field-grown material was validated on the spectral data of an additional set of independent sweet-seed and bitter-seed genotypes grown in greenhouse conditions in a different year. The discrimination ability was assessed by measuring (a) the rate of misclassified observations; (b) the Sensitivity of the model (as the ratio of correctly predicted samples in a class relative to the actual samples belonging to that class); (c) the Specificity of the model (as the ratio of samples not predicted to be in a class relative to the actual samples not belonging to that class); and (d) the classification error according to the following formula:

1 − [(Sensitivity + Specificity)/2].

## Figures and Tables

**Table 1:** Mean value (mg/kg) and proportion, and range values, for total quinolizidine alkaloid (QA) content and content of 13 alkaloid compounds in 142 broadly sweet-seed breeding lines and 45 landrace accessions of white lupin.

	Broadly Sweet-Seed Lines			Bitter-Seed Landrace Accessions		
Variable	Mean Value	Mean Proportion (%)	Range Values	Mean Value	Mean Proportion (%)	Range Values
Total QA content	338.3	−	94.9–990.4	25,613.2	−	14,041.2–37,321.4
Lupanine	208.1	61.6	38.5–651.3	20,855.7	81.4	11,326.4–32,297.3
13α-hydroxylupanine	32.5	9.6	0.0–139.1	418.6	1.6	57.0–1280.5
13α-angeloyloxylupanine	28.5	8.4	0.0–126.6	153.9	0.6	51.4–496.2
Angustifoline	12.2	3.6	1.2–45.9	369.2	1.4	106.4–654.9
N-methylalbine	12.0	3.6	1.0–62.5	338.8	1.3	50.0–1601.1
α-isolupanine	11.3	3.3	2.8–29.2	145.8	0.6	66.8–264.6
Ammodendrine	11.2	3.3	3.0–27.5	120.0	0.5	46.4–350.2
13α-tigloyloxylupanine	8.7	2.6	0.0–28.6	18.6	0.1	3.4–50.0
Multiflorine	5.1	1.5	1.0–29.5	2377.2	9.3	218.2–5011.8
Tetrahydrorhombifoline	4.7	1.4	1.0–17.3	195.5	0.8	35.6–449.6
17-oxolupanine	2.1	0.6	0.0–13.4	143.2	0.6	32.4–582.4
13-hydroxymultiflorine	1.2	0.4	0.0–7.6	24.1	0.1	7.1–50.1
Albine	0.8	0.2	0.0–5.3	452.2	1.8	101.3–1206.6

**Table 2:** Number of landrace accessions, and mean and range values of total quinolizidine alkaloid content (mg/kg), for 11 regional germplasm pools of white lupin.

Germplasm Pool	Number	Mean ^a^	Range
Spain	4	32,701.5 a	31,056.8–34,502.1
Greece	4	32,078.4 a	25,588.9–37,321.4
Canary Islands	4	30,260.1 a	27,832.0–32,057.9
Azores	4	27,285.2	23,903.7–29,555.1
Portugal	4	26,110.6	20,686.6–30,291.3
Maghreb	4	25,680.4	22,024.5–27,425.5
Egypt	2	25,310.4 b	22,663.9–27,956.8
Turkey	4	24,280.8 b	20,616.7–30,361.6
East Africa	3	22,682.9 b	18,007.9–27,461.6
Italy	8	20,341.5 b	14,041.2–27,909.4
Near East	4	19,401.1 b	15,776.9–22,149.3
LSD (*p* < 0.05)	−	5453.8	−

^a^ Values followed by letter ‘a’ and ‘b’ do not differ from the top-ranking and bottom-ranking value, respectively, at *p* < 0.05 (error term = landrace variation within germplasm pool).

**Table 3:** Performances of the best NIRS-based models in quantifying white lupin total quinolizidine alkaloid content. LV: number of latent variables; SEP: Standard Error of Prediction (for repeated double cross-validation); RPD: Ratio of standard error of Prediction to standard Deviation.

Material	Samples	LV	SEP	*R* ^2^	RPD
Broadly sweet lupins	Whole seeds	1	179.6 mg/kg	0.038	1.02
Broadly sweet lupins	Flours	2	164.1 mg/kg	0.088	1.05
Bitter lupins	Whole seeds	6	5598.8 mg/kg	0.293	1.19
Bitter lupins	Flours	3	4122.9 mg/kg	0.467	1.37
Broadly sweet-seed + bitter lupins	Whole seeds	6	0.260 Log(mg/kg)	0.889	3.00
Broadly sweet-seed + bitter lupins	Flours	4	0.209 Log(mg/kg)	0.932	3.84

## Data Availability

The evaluation data are available in the Supplementary Materials.

## Supplementary Materials

The following supporting information can be downloaded at https://www.mdpi.com/article/10.3390/plants14213327/s1. Table S1: Total quinolizidine alkaloid (QA) content of 45 white lupin landrace accessions of different geographic origin. Table S2: Ability in validation data sets of Partial Least Squares Discriminant Analysis models to discriminate broadly sweet-seed and bitter-seed white lupin material for (a) whole seeds, averaged spectra of 10 seeds; (b) flour spectra; and (c) whole seeds, spectra of individual seeds. Figure S1: Complete set of recorded spectra obtained from individual whole seed (left) and flour (right) samples. Data set plants-14-03327.xls: QA data used for the study.

## Abbreviations

The following abbreviations are used in this manuscript:

GC/FID	Gas Chromatography with Flame Ionization Detector
GC/MS	Gas chromatography–mass spectrometry
LV	Latent variables
NIRS	Near-Infrared Spectroscopy
RPD	Ratio of standard error of Prediction to standard Deviation
