# Supplementary material for: Quinolizidine Alkaloid Composition of White Lupin Landraces and Breeding Lines, and Near-Infrared Spectroscopy-Based Discrimination of Low-Alkaloid Material
Source: Plants (Basel). 2025 Oct 30;14(21):3327. doi: 10.3390/plants14213327 (PMC12608174; doi:10.3390/plants14213327)

**Supplementary Table S1** Total quinolizidine alkaloid (QA) content of 45 white lupin landrace accessions of different geographic origin

| Accession | Origin | Total QA (mg/kg) |
| --- | --- | --- |
| Gr56 | Greece | 37321.4 |
| E3 | Spain | 34502.1 |
| E16 | Spain | 33633.4 |
| Gr05 | Greece | 33304.6 |
| Gr33 | Greece | 32098.6 |
| La642 | Canary Islands | 32057.9 |
| E36 | Spain | 31613.6 |
| La648 | Canary Islands | 31440.2 |
| E2 | Spain | 31056.8 |
| La431 | Turkey | 30361.6 |
| E107 | Portugal | 30291.3 |
| La653 | Canary Islands | 29710.4 |
| Ac155 | Azores | 29555.1 |
| Ac050 | Azores | 29353.8 |
| Egy76 | Egypt | 27956.8 |
| La246 | Italy | 27909.4 |
| La646 | Canary Islands | 27832.0 |
| La654 | Kenya | 27461.6 |
| La686 | Algeria | 27425.5 |
| Maroc78 | Morocco | 27088.9 |
| E126 | Portugal | 26803.9 |
| E132 | Portugal | 26660.5 |
| Ac139 | Azores | 26328.0 |
| La150 | Morocco | 26183.0 |
| Gr57 | Greece | 25588.9 |
| La120 | Turkey | 23936.4 |
| Ac085 | Azores | 23903.7 |
| Ita49 | Italy | 23241.8 |
| Egy64 | Egypt | 22663.9 |
| La020 | Ethiopia | 22579.2 |
| Ita57 | Italy | 22214.2 |
| La110 | Turkey | 22208.7 |
| La415 | Jordan | 22149.3 |
| Maroc74 | Morocco | 22024.5 |
| LAP123 | Italy | 21229.1 |
| LAP124 | Italy | 20749.2 |
| E91 | Portugal | 20686.6 |
| La259 | Turkey | 20616.7 |
| La406 | Israel | 20111.5 |
| La427 | Syria | 19566.5 |
| LAP108 | Italy | 18367.2 |
| Ethiop98 | Ethiopia | 18007.9 |
| La673 | Lebanon | 15776.9 |
| Ita1 | Italy | 14980.1 |
| LAP122 | Italy | 14041.2 |

**Supplementary Table S2** Ability in validation data sets of Partial Least Squares Discriminant Analysis models to discriminate broadly sweet-seed and bitter-seed white lupin material, for (a) whole seeds, averaged spectra of 10 seeds, (b) flour spectra, and (c) whole seeds, spectra of individual seeds

|  | **Whole seeds, averaged spectra** | **Flour spectra** | **Whole seeds, individual spectra** |
| --- | --- | --- | --- |
| Number of spectra | 93 | 92 | 910 |
| Misclassified observations (%) | 0 | 0 | 0.88 |
| Sensitivity | 1.000 | 1.000 | 0.978 |
| Specificity | 1.000 | 1.000 | 0.997 |
| Classification error | 0 | 0 | 0.013 |

**Supplementary Figure S1** Complete set of recorded spectra obtained from individual whole seed (left) and flour (right) samples


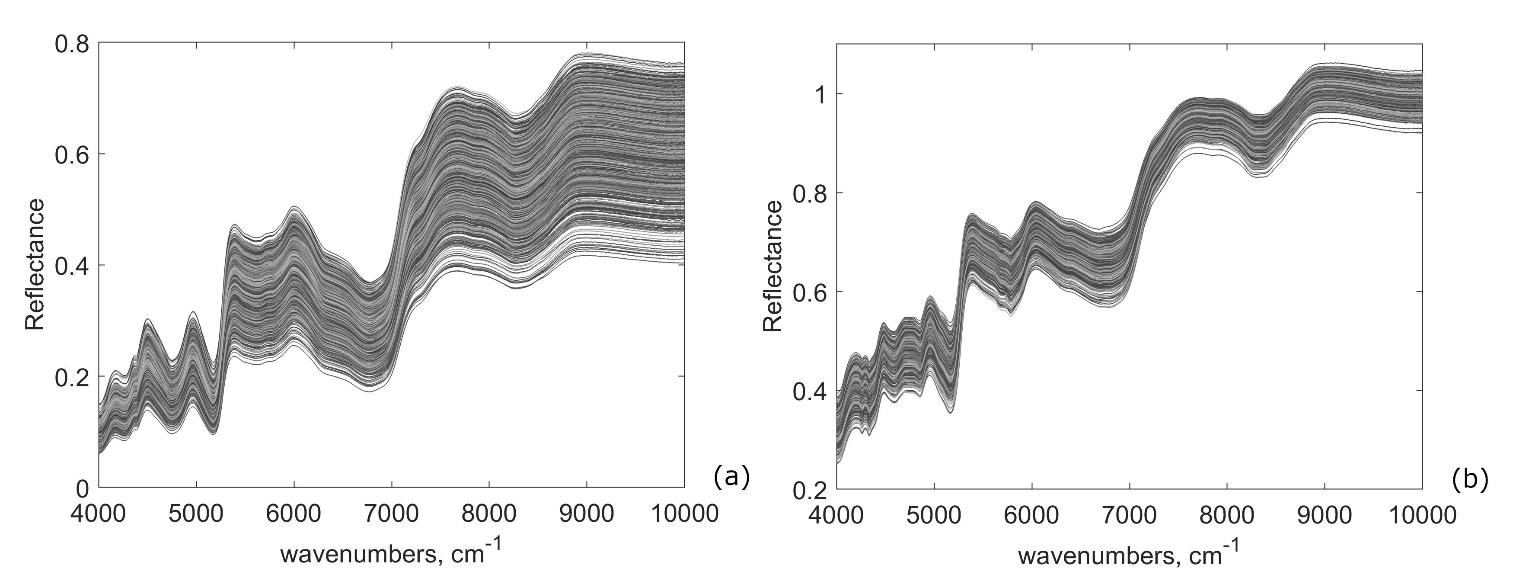

Supplement: Supplementary file 1 [file plants-14-03327-s001.zip › plants-3808086_supplementary_material.docx]
